# The Humboldt Medical Archives

**Published:** 1867-09

**Authors:** 


THE HUMBOLDT MEDICAL ARCHIVES.—We are in receipt of the first number of this journal. It is under the editorial charge of Drs. A. Hammer and M. A. Pallen, and the faculty of the Humboldt Medical College of St. Louis as co-editors. It is a monthly of sixty pages and presents a fine typographical appearance.

